# Early development of bacterial community diversity in emergently placed urinary catheters

**DOI:** 10.1186/1756-0500-5-332

**Published:** 2012-06-27

**Authors:** Betsy Foxman, Jianfeng Wu, Emily C Farrer, Deborah E Goldberg, John G Younger, Chuanwu Xi

**Affiliations:** 1 Department of Epidemiology, University of Michigan School of Public Health, 1415 Washington Heights, Ann Arbor, MI 48109-2029, USA; 2 Department of Environmental Health Sciences, University of Michigan School of Public Health, Ann Arbor, MI, USA; 3 Department of Ecology and Evolutionary Biology, University of Michigan College of Literature, Science & the Arts, Ann Arbor, MI, USA; 4 Department of Emergency Medicine, University of Michigan School of Medicine, Ann Arbor, MI, USA

**Keywords:** Urinary tract infection, Microbial ecology, Biofilms, Urinary catheter

## Abstract

**Background:**

Approximately 25% of hospitalized patients have a urinary catheter, and catheter associated urinary tract infection is the most common nosocomial infection in the US, causing >1 million cases/year. However, the natural history of the biofilms that rapidly form on urinary catheters and lead to infection is not well described.

**Findings:**

We characterized the dynamics of catheter colonization among catheters collected from 3 women and 5 men in a trauma burn unit with different indwelling times using TRFLP and culture. All patients received antibiotic therapy. Results: Colony-forming units increased along the extraluminal catheter surface from the catheter balloon to the urethra, but no trend was apparent for the intraluminal surface. This suggests extraluminal bacteria come from periurethral communities while intraluminal bacteria are introduced via the catheter or already inhabit the urine/bladder. Richness of operational taxonomic units (OTUs) increased over time on the intraluminal surface, but was constant extraluminally.

**Conclusions:**

OTU community composition was explained best by time rather than axial location or surface. Our results suggest that catheter colonization can be very dynamic, and possibly have a predictable succession.

## Findings

### Background

Catheter associated urinary tract infection is the most common nosocomial infection, causing >1 million cases annually in the United States [1]. All types of indwelling urinary catheters, including silver-coated and those impregnated with antibiotics, are colonized by biofilms -- an essential pathogenic feature of catheter infections [2]. Biofilms tend to be resistant to antibiotic treatment and may enhance the development of further antibiotic resistance [3]. The majority (88%) of urinary catheter biofilms contain 3 or more microbial species [4]. It is assumed that a biofilm on a catheter pre-disposes to bacteriuria, but it is uncertain if the biofilm is the source of bacteriuria [5]. Among catheterized patients, the risk of bacteriuria is ~ 3-8% per day. Almost all will be colonized after one month [6].

Bacteria are introduced with the urinary catheter, or ascend the extraluminal surface from the urethra to the bladder after the catheter is inserted [7]. Intraluminal colonization can occur if there are breaks in infection control, leading to contamination of the catheter bag, or failure of the closed drainage system [8]. Information on the bacterial community structure and dynamics of catheter biofilm development is very limited. In a study of 34 urology patients, Barford et al. [7] showed the number of colony forming units (CFUs) recovered from the urinary catheter surface increases with duration of catheterization, and the number of CFUs increases more rapidly on the extraluminxal than intraluminal surface. Barford et al. [7] and a study by Frank et al. [9], following 14 patients catheterized for two weeks following total prostatectomy, also reported on differences in community structure between catheter intraluminal and extraluminal surfaces, finding more different strains on extraluminal than intraluminal surfaces. Frank et al. further reported that each catheter had unique biofilm communities and intraluminal and extraluminal communities on a given catheter were often substantially different [9]. The faster colonization on the extraluminal than intraluminal surface, and its distinct composition, suggests that the extraluminal surface is inoculated from the periurethral skin upon insertion and the intraluminal surface is either colonized by existing bladder inhabitants or those that colonize the urine following catheter insertion [2]. Presumably an analogous mechanism occurs in other devices, such as percutaneous nephrostomy tubes and urethral stents, placed in the upper urinary tract. With the exception of Barford et al. [7], we found no studies actually following the dynamics of catheter biofilms over time, and the dynamics in Barford et al. are limited to changes in total CFUs and not community composition. Understanding biofilm dynamics is essential for identifying ways to manipulate biofilm growth on catheters and prevent catheter-associated infection.

We used terminal restriction fragment length polymorphism analysis (TRFLP) and CFU counts to characterize indwelling urinary catheter microbial community dynamics as a function of catheterization duration, intra- or extraluminal device surface, and distance from the catheter balloon to the external environment. TRFLP is a method for profiling microbial communities based on DNA [10]. We were particularly interested in whether a predictable succession in microbial community structure was observed and whether differences in dynamics between catheter surfaces and distance from the catheter balloon could be used to infer the sources of the microbes colonizing the catheter.

### Materials and methods

#### Study population

We collected urinary catheters from a convenience sample of adult polytrauma patients with multisystem injury without hemodynamic instability with or without instability or shock resuscitated in the emergency department of a Level I trauma center and admitted to the trauma and burn intensive care unit. Catheters were inserted using aseptic procedures including cleaning of the urethral meatus with a betadyne solution. All catheters were silver impregnated. Catheters were collected for analysis when the treating team believed it appropriate for their removal. Catheters were transferred in sterile containers to the laboratory, and refrigerated until processing, which occurred within 1 hour following removal. No modifications to standard critical care practice were imposed. Participants were likely treated with antibiotics during their hospital stay, but we did not have access to that information. The proposed use of catheters and urine was reviewed by the University of Michigan institutional review committee on human subjects protection and considered exempt because we used discarded materials that were not identifiable.

#### Sample collection and treatment

Catheters were marked, at the time of removal, to indicate the point of entry into the urethra. Catheters collected from male patients were cut into four axial segments. The most proximal ‘bladder’ section included the inserted tip of the catheter and the associated balloon. Sections 2 and 3 were subsequent 10 cm segments comprising the ‘deep urethral’ and ‘superficial urethral’ regions. The fourth segment was an additional 10 cm ‘external’ segment. Catheters collected from female patients were divided into a bladder segment, a 10 cm urethral segment, and an external segment. Each section was bisected lengthwise to allow sampling of the intraluminal and extraluminal device surfaces separately, thus organizing each device into 8 and 6 spatial zones for male and female patients, respectively. Bacteria were collected from each zone using a sterile cotton swab which was placed into 2 ml of 1 x PBS buffer, then recovered using the Swab Extraction Tube System (Fisher Scientific). Cells were resuspended in 1 ml of PBS. Urine (20 mL) was collected from the catheter port of 5 participants (three male patients, two catheterized for one day, and one for two days; and two female patients, one catheterized for two days, the other for nine days) at time of catheter removal. Urine was centrifuged at 10,000× g for 10 min at 4 °C and the pellets were resuspended in 1 ml of PBS. 100 μl of cell suspensions from the catheters and urine was used for quantitative culture on tryptic soy agar and blood agar. The plates were incubated at 37 °C for two days or until colonies showed. The remaining 900 μl of cell suspension from the catheters was used for DNA extraction. For the analysis, the number of CFUs for the two plates were averaged.

#### Terminal restriction fragment length polymorphism (TRFLP) analysis

Bacterial community fingerprints were obtained using TRFLP [10]. DNA was extracted from bacterial cells from urine samples and each catheter zone using the MasterPure™ DNA Purification Kit (Epicentre Biotechnologies, Madison, WI). Polymerase chain reaction (PCR) amplification was performed in triplicate with the primer pairs 63F: 5-CAGGCCTAACACATGCAAGTC -3′ and 1389R: 5- ACGGGCGGTGTGTACAAG -3′ targeting general bacterial 16S rRNA gene [11]. The forward primer was 5’-labeled with the fluorescent dye 6-FAM (6-carboxyfluorescein) (IDT, Coralville, IA). To minimize amplification of chimeras and pseudo-terminal restriction fragments, 25 cycles were conducted with extended elongation time of 3 minutes [12]. After amplification, the triplicate PCR products were pooled and purified using a QIAquick PCR purification kit (Qiagene, Valencia, CA). The purified PCR products were digested with restriction enzymes *Hha* I or *Msp* I (NEB, Ipswich, MA) for 4 hours at 37°C. Following digestion, samples were loaded into a 96-well GS1000 ROX1000 CE plate and sent to the DNA Core at University of Michigan for T-RFLP analysis. Peaks in each profile were related to specific fragment lengths based on a size marker (50-1000 MapMarker, GeneScan™, Applied Biosystems). Data were retrieved using Peak Scanner software v1.0 (Applied Biosystems) and normalized using T-Align [13] before further statistical analysis. Each distinct 16s fragment length produced was considered an operational taxonomic unit (OTU).

#### Statistical analyses

For number of CFUs and OTU richness, the effects of time, axial location, and intraluminal/extraluminal surface type, as well as interactions between axial location, time, and surface type were tested with a repeated-measures, mixed-effects model in R [14]. CFU was log transformed prior to analysis. For OTU richness, because two different digests were performed, both restriction digests were combined in one model and digest was included as a main effect. Study subject was modeled as a random effect. For all analyses, the significance of fixed effects (factors of interest that are not a random sample from a larger population) was tested with F tests, and the significance of random effects (factors whose levels are a random sample from a larger population) and the proper covariance structure for the repeated measures were tested using likelihood ratio tests [15]. In this study of dynamics, we treated time as a fixed factor because it was the central factor of interest. Axial location was modeled as a continuous variable (1, 2, 3, 4 for males and 1, 2, 3 for females), because AIC scores were lower for both CFU and OTU richness when models with axial location as a continuous vs. a categorical (3 categories, balloon, middle, and air) variable were compared.

To analyze whether subject, time, axial location, and surface affected OTU community composition, we calculated pairwise Bray-Curtis dissimilarity indices among all samples separately for each of the *Hha* I and *Msp* I digests. Bray-Curtis ranges between 0 and 1: a value of 0 indicates samples have the same species composition and 1 indicates samples have no species in common. We used the quantitative form of Bray-Curtis which takes into consideration amount, not just presence/absence, of each OTU: $\text{djk}=\sum \left|\text{xij}-\text{xik}\right|/\sum \left(\text{xij}+\text{xik}\right)$, where dj,k is the dissimilarity between samples j and k, and xi is the abundance (standardized peak area) of OTU i. This resulted in one dissimilarity matrix for each digest. We then tested the significance of subject, time, length, and surface for degree of community dissimilarity using permutation tests in R for each digest (package vegan, functions vegdist and adonis). Because subject and time were completely confounded (each subject was sampled once, at a given time) and because adonis and other multivariate permutation programs do not have the capacity for complex random effects structure in the analysis, we first tested the effect of subject with a model including only subject as the independent variable. We then tested the effect of time in a model including only time as the independent variable. Lastly, we tested the effect of axial location and surface in the same model with permutations restricted within subjects.

To visualize OTU community composition for the *Hha* I digest, an NMDS (non-metric multi-dimensional scaling) plot was constructed based on the Bray-Curtis dissimilarity matrix. For ease of visualization, for each subject, all samples from the extraluminal surface were combined and all samples from the intraluminal were combined. For five of the subjects, urine samples were also obtained; these samples were included in the NMDS.

### Results

Urinary catheters placed as part of the resuscitation of acutely injured outpatients were collected from 3 women and 5 men, and had been in place an average of 3 days (range 1 to 16) before removal.

#### Colony forming units on tryptic soy and blood agars

**Figure 1:**
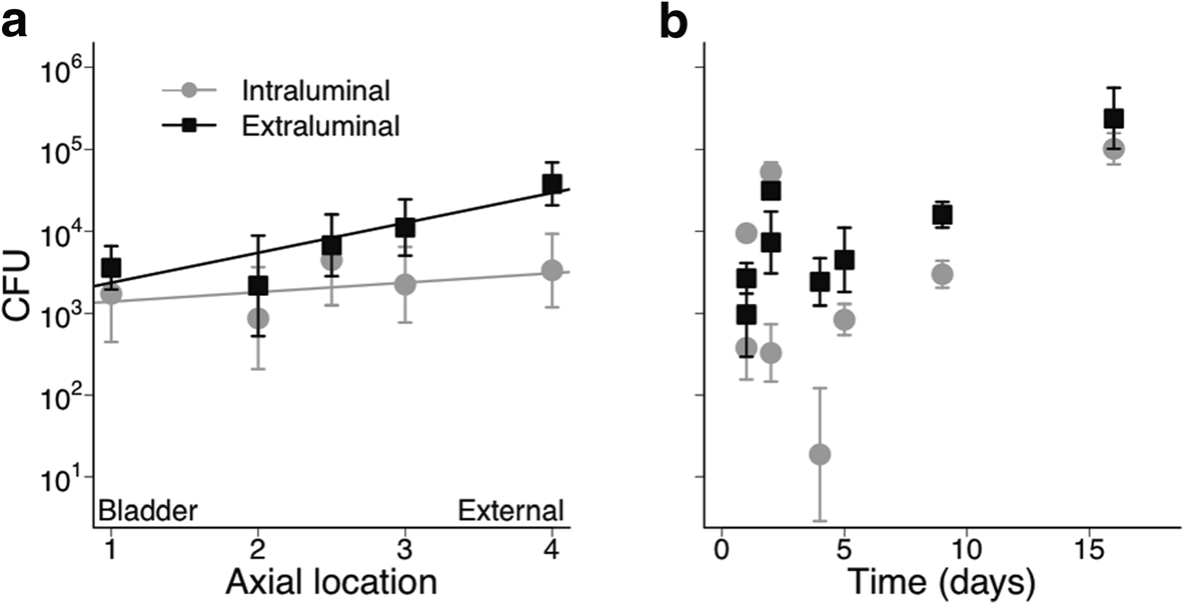
**Number of colony forming units (CFU) in the intraluminal (gray) and extraluminal (black) surfaces along the length of the urinary catheter (a) and over time (b).** For (**a**), each point represents the mean and standard error over all individuals for each axial location of the catheter (males had 4 distinct locations, females had 3 locations). For (**b**), points represent mean and standard error for each surface of each individual. Note that the y-axis is on a log scale. Lines indicate a significant or nearly significant relationship based on the parameter estimates from mixed model analysis; for P values see Table 1. Note that the means depicted do not incorporate subject effects. Urinary catheters from 5 males and 3 females catheterized in a level 1 trauma center for 1 to 16 days.

The number of CFUs cultured from the catheters varied greatly, with a 200-fold difference between the lowest and highest totals across subjects (Additional file 1: Table S1). Nevertheless, on average, the number of CFUs significantly increased from the bladder to the external segment (Table 1, Figure 1a). Although not quite statistically significant, there was a strong suggestion that this effect was limited to the extraluminal surface (p = 0.053 for interaction between surface and axial location). There was no detectable statistical association between duration of catheter placement and recovered CFUs (Figure 1b, Table 1). However, we had more statistical power to detect differences among axial locations (Figure 1a) than with duration of catheter placement (Figure 1b), because the statistical tests for surface and axial location take into account patterns within each subject, while we had only a single subject for each duration, so the trend apparent in Figure 1b may be of biological significance.

**Table 1:** Effect of time, extra- versus intraluminal location, and axial location on number of colony forming units (CFU) in urinary catheters

	**ndf**	**ddf**	**F**	**P**
Fixed effects				
Time	1	6	3.69	0.1033
Surface	1	46	0.02	0.8952
Axial location	1	46	14.69	0.0004***
Surface × time	1	46	0.03	0.8682
Surface × axial location	1	46	3.95	0.0528†
		df	LR statistic	P
Random effects				
Subject, intercept		0, 1	20.6	<0.0001***

Axial location was modeled as a continuous variable (bladder = 1,deep urethral = 2, superficial urethral = 3, external = 4 for males and bladder = 1, urethral = 2.5, and external = 4 for females). The significance of random effect (subject) and the proper covariance structure for the repeated measures were tested using likelihood ratio tests. Ndf is numerator degrees of freedom, ddf is denominator degrees of freedom. Urinary catheters from 5 males and 3 females catheterized in a level 1 trauma center for 1 to 16 days.

*** is p <0.001 and †p <0.10.

#### OTU richness

The two digests, *Hha* I and *Msp* I, differed significantly in the number of distinct OTUs they produced (Table 2) with *Hha* I having fewer than *Msp* I (76 and 91 OTUs, respectively). Subjects also differed in the OTU richness found per catheter (Table 2), ranging from 15-36 OTUs for the *Hha* I digest and 13-44 OTUs for the *Msp* I digest. Unlike total recovered CFU counts, OTU richness differed between surfaces over time, with the intraluminal surface accumulating OTUs over time and the extraluminal surface staying more or less similar in richness over time ( Additional file 1: Figure S2, significant surface x time interaction in Table 2).

**Table 2:** Effects of digest, time, surface, and axial location on OTU richness in urinary catheters

	**ndf**	**ddf**	**F**	**P**
Fixed effects				
Digest	1	100	5.92	0.0168*
Duration	1	6	1.31	0.2959
Surface	1	100	0.43	0.5119
Axial location	1	100	1.70	0.1949
Surface × duration	1	100	5.41	0.0220*
Surface × axial location	1	100	2.41	0.1234
		df	LR statistic	P
Random effects				
Subject, intercept		0, 1	16.7	<0.0001***

Axial location was modeled as a continuous variable (1, 2, 3, 4 for males and 1, 2.5, 4 for females). The significance of random effect (subject) and the proper covariance structure for the repeated measures were tested using likelihood ratio tests. Ndf is numerator degrees of freedom, ddf is denominator degrees of freedom. Urinary catheters from 5 males and 3 females catheterized in a level 1 trauma center for 1 to 16 days.

*** is p <0.001, *p <0.05.

#### OTU community composition

We calculated Bray-Curtis dissimilarity indices among samples to assess differences in OTU community composition on the catheters, and used permutation tests to test significance of explanatory variables. Subjects differed in community composition and relative abundance for both *Hha* I and *Msp* I digests (Table 3), i.e., pairwise comparisons of samples taken within subjects were more similar than pairwise comparisons among subjects (data not shown). For the *Hha* I digest, community composition shifted over time in ways that seemed to be consistent among subjects, so that communities were more similar among patients who had catheters for similar amounts of time than among patients with very different durations of catheterization (significant effect of time in Table 3, Additional file 1: Figure S2). Degree of similarity among subjects seemed to depend on time; communities were more similar among subjects with catheters in place for longer periods of time ( Additional file 1: Figure S2). In addition, OTU composition tended to differ (although not significantly) between the intraluminal and extraluminal surfaces for the *Hha* I digest (Table 3). For both digests, OTU community composition changed over catheter length, so that proximal axial locations more similar among subjects than more distant axial locations (data not shown).

**Table 3:** Analyses of variance using community dissimilarity matrices with permutation tests for significance (function adonis in R package vegan) for digests from microbial communities from urinary catheters

**Digest**	**F**	**R**^**2**^	**P**
Hha1			
Subject	4.558	0.3943	<0.001***
Time	5.168	0.0859	<0.001***
Surface	1.247	0.0220	0.0728†
Axial location	1.404	0.0248	0.0443*
Msp1			
Subject	13.0268	0.6551	<0.001***
Time	0.7831	0.0143	0.4522
Surface	0.3593	0.0066	0.4373
Axial location	0.8751	0.0161	0.0483*

For each digest, results are given from three different analyses: the effect of subject was tested in a model with subject as the only independent variable and the effect of time was tested in a model with time as the only independent variable. Surface and axial location (continuous) were tested together in the same model, with permutation tests restricted within subject in a type III anova. Digests of microbial communities from urinary catheters from 5 males and 3 females catheterized in a level 1 trauma center for 1 to 16 days.

*** is p <0.001, *p <0.05 and †p <0.10.

### Comment

Culture and TRFLP analysis of 8 urinary catheters placed for acute trauma resuscitation and left in place between 1 and 16 days showed rapid development of complex microbial community structures that varied by location within the device. The key findings of the current report are these. First, the cultivable burden of organisms attached to the devices was higher on the extraluminal compared to intraluminal surface particularly toward the external portion of the catheter (Figure 1). The number of colony-forming units tended to increase from the bladder to outside the body on the extraluminal surface while remaining similar along the inner surface. This is not surprising given the differences in potential sources of colonists found along the extraluminal surface (from bladder to urethra to patient bed) and the more similar potential sources along the intraluminal surface (urine), and that the urine likely included antibiotics. However, it is possible that - despite using the same methods - that recovery was greater on the extraluminal surface, or that the external catheter surface was contaminated during catheter removal. We believe that unlikely, as catheters were processed rapidly following removal, and our findings are consistent with other, similar studies. In a study of catheters from 34 urology patients all segments of the extraluminal surface were colonized by day 1 but it was not until day 4 that all axial locations along the intraluminal surfaces were colonized [7].

Second, using non-culture methods, we observed an increase in the richness of OTUs over time on the intraluminal surface, but constant richness extraluminally and with axial location. The OTU composition of the communities changed linearly over time, but surface had only marginal effects on the OTU community composition. This contrasts with Barford et al.’s study of 34 urology patients, which found significantly more strains and genera on extraluminal than intraluminal surfaces, although they did not present data by time since catheterization [7]. We found no other reports for comparison. Both our and Barford’s results are consistent with the hypothesis that the extraluminal surface is inoculated during catheter insertion.

Finally, results from our analysis of species composition on the catheters suggest that there may be a predictable succession in microbial communities over time ( Additional file 1: Figure S2). We found no similar analyses in the literature to support or refute this observation. Most catheter-associated infections of the urinary tract are asymptomatic and do not progress to symptomatic infection, but colonization is the first step towards symptomatic infection [4]. An outstanding question - which we could not directly address - is the extent to which the catheter biofilm is the source of asymptomatic bacteriuria and catheter-associated urinary tract infection. A study of 86 patients with urethral catheters in Japan, found that urinary and intraluminal catheter isolates were not always the same, independent of antibiotic therapy [6]. A 1999 study of 1,497 newly catheterized patients conducted at Mayo Clinic compared paired daily urine cultures from the catheter specimen port and collection bag, and assumed that extraluminal infections would be first detected in the specimen port and intraluminal infections in the collection bag. They concluded that 66% of infections were extraluminally acquired [16]. However, they assumed that intraluminal infections were caused by microorganisms ascending the catheter, an assumption not supported by our study or that of Barford et al. [7] which both suggest that bacteria colonizing the intraluminary surface of the catheter and urine have a common source. Further, for the 5 urine samples we tested, the urinary communities tended to be more similar to intraluminal than extraluminal communities (Bray-Curtis dissimilarity of 0.334 (SE 0.018) vs. 0.435 (SE 0.041)). It is possible that the bacteria that grow best on catheter surfaces are not the same as those that grow well in planktonic form in the urine or that bind well to urinary tract tissues thereby interacting with the host to cause infection. Notably, in a study of 14 patients catheterized for 2 weeks following total prostatectomy, the most prevalent species found in catheter biofilms (either surface) were *Pseudomonas aeruginosa* (64%), *Klebsiella pneumoniae* (63%) and *Escherichia coli* (50%). The bacterial species that most often cause catheter-associated UTI is *Escherichia coli*[4]. As bacteriuria from catheterized patients is often polymicrobial - especially in patients where catheterization is of longer duration - it is also possible that the microbial community structure may mediate interaction with the host.

### Conclusions

This pilot study is a first step toward understanding the dynamics of catheter colonization. Like all studies conducted in real populations, there were inevitable compromises in data collection: we had no control over the treatment of patients with antibiotics, time of catheter removal, and reason for hospitalization. Nonetheless, we detected significant trends and patterns that are unlikely to be the result of systematic error. Future, larger, studies, with greater resolution of species present, are needed to identify how changing microbial communities are related to the development of asymptomatic bacteriuria and symptomatic infection.

## Competing interests

The authors declare that they no competing interests.

## Authors’ contribution

BF participated in study design and data analysis and drafted the manuscript, JW conducted the laboratory experiments and associated data analyses, EF performed the data analysis, DG participated in study design, and analysis and interpretation of data, JY participated in study design, oversaw data collection and participated in analysis and interpretation of data, CX participated in study design, oversaw laboratory analysis, and participated in analysis and interpretation of data. All authors read and approved the final manuscript.

## Supplementary Material

Additional file 1 **Table S1.** Cultivable bacteria on each axial location of urinary catheters (CFU/part or CFU/ml for urine samples). Urinary catheters from 5 males (labeled Ma to Me) and 3 females (labeled Fa to Fc) catheterized in a level 1 trauma center for 1 to 16 days. Data are given for the luminal and extraluminal surfaces, each divided into three (female) or four (male) axial locations from internal (bladder) to external (exposed to air), as well as for urine samples for a subset of subjects. **Figure S1**. OTU richness in intraluminal and extraluminal surfaces over time for the *Hha* I (A) and *Msp* I (B) digests. Each point represents the mean and standard error over the length of the catheter for each surface of each individual. Lines indicate a significant or nearly significant relationship; for P values from mixed model analysis of both digests see Table 2. Urinary catheters from 5 males and 3 females catheterized in a level 1 trauma center for 1 to 16 days. **Figure S2**. Non-metric multi-dimensional scaling (NMDS) ordination of the OTU community from the *Hha* I digest based on Bray-Curtis dissimilarity index. Degree of shading indicates the length of time the catheter was in place, white = 1 day to black = 16 days. Males are represented by squares and solid lines, and females by circles and dashed lines. I is the intraluminal surface, E is the extraluminal surface, and U is urine. Lines connect samples from the same subject. For each subject, all samples from intraluminal and all samples from extraluminal surfaces were combined. Note that samples from catheters that were in place for longer periods of time are clustered and have lower variability compared to samples from catheters in place for shorter periods of time (significant effect of time, Table 2). Also note that the urine samples tend to be more similar (closer) to the intraluminal samples than the extraluminal samples from each subject. Digest of microbial communities from urinary catheters from 5 males and 3 females catheterized in a level 1 trauma center for 1 to 16 days. Samples from the *MSP* I digest showed similar patterns. Click here for file

## References

[B1] Klevens RM Edwards JR Richards CL Estimating health care-associated infections and deaths in U.S. hospitals, 2002 Public Health Rep 2007 122 2 160 166 17357358 10.1177/003335490712200205 PMC1820440

[B2] Stickler DJ Bacterial biofilms in patients with indwelling urinary catheters Nat Clin Pract Urol 2008 5 11 598 608 18852707 10.1038/ncpuro1231

[B3] Francolini I Donelli G Prevention and control of biofilm-based medical-device-related infections FEMS Immunol Med Microbiol 2010 59 3 227 238 20412300 10.1111/j.1574-695X.2010.00665.x

[B4] Hola V Ruzicka F Horka M Microbial diversity in biofilm infections of the urinary tract with the use of sonication techniques FEMS Immunol Med Microbiol 2010 59 3 525 528 20602639 10.1111/j.1574-695X.2010.00703.x

[B5] Matsukawa M Kunishima Y Takahashi S Bacterial colonization on intraluminal surface of urethral catheter Urology 2005 65 3 440 444 10.1016/j.urology.2004.10.065 15780351

[B6] Hooton TM Bradley SF Cardenas DD Diagnosis, prevention, and treatment of catheter-associated urinary tract infection in adults: 2009 International Clinical Practice Guidelines from the Infectious Diseases Society of America Clin Infect Dis 2010 50 5 625 663 10.1086/650482 20175247

[B7] Barford JM Anson K Hu Y Coates AR A model of catheter-associated urinary tract infection initiated by bacterial contamination of the catheter tip BJU Int 2008 102 1 67 74 10.1111/j.1464-410X.2008.07465.x 18284413

[B8] Saint S Chenoweth CE Biofilms and catheter-associated urinary tract infections Infect Dis Clin North Am 2003 17 2 411 432 Review 10.1016/S0891-5520(03)00011-4 12848477

[B9] Frank DN Wilson SS St Amand AL Culture-independent microbiological analysis of foley urinary catheter biofilms PLoS One 2009 4 11 e7811 10.1371/journal.pone.0007811 19907661 PMC2771765

[B10] Liu WT Marsh TL Cheng H Characterization of microbial diversity by determining terminal restriction fragment length polymorphisms of genes encoding 16S rRNA Appl Environ Microbiol 1997 63 11 4516 4522 9361437 10.1128/aem.63.11.4516-4522.1997 PMC168770

[B11] Osborn AM Moore ER Timmis KN An evaluation of terminal-restriction fragment length polymorphism (T-RFLP) analysis for the study of microbial community structure and dynamics Environ Microbiol 2000 2 1 39 50 10.1046/j.1462-2920.2000.00081.x 11243261

[B12] Egert M Friedrich MW Formation of pseudo-terminal restriction fragments, a PCR-related bias affecting terminal restriction fragment length polymorphism analysis of microbial community structure Appl Environ Microbiol 2003 69 5 2555 2562 10.1128/AEM.69.5.2555-2562.2003 12732521 PMC154551

[B13] Smith CJ Danilowicz BS Clear AK T-Align, a web-based tool for comparison of multiple terminal restriction fragment length polymorphism profiles FEMS Microbiol Ecol 2005 54 3 375 380 10.1016/j.femsec.2005.05.002 16332335

[B14] R Development Core Team R: A language and environment for statistical computing 2008 Vienna: R Foundation for Statistical Computing

[B15] West BT Welch KB Galecki AT Linear mixed models: a practical guide using statistical software 2007 Boca Raton: Chapman & Hall/CRC Press

[B16] Tambyah PA Halvorson KT Maki DG A prospective study of pathogenesis of catheter-associated urinary tract infections Mayo Clin Proc 1999 74 2 131 136 10.4065/74.2.131 10069349

