# Supplementary material for: Early development of bacterial community diversity in emergently placed urinary catheters
Source: BMC Res Notes. 2012 Jun 27;5:332. doi: 10.1186/1756-0500-5-332 (PMC3500218; doi:10.1186/1756-0500-5-332)

SUPPLEMENTAL MATERIAL

Table S1. Cultivable bacteria on each axial location of urinary catheters (CFU/part or CFU/ml for urine samples). Urinary catheters from 5 males (labeled Ma to Me) and 3 females (labeled Fa to Fc) catheterized in a level 1 trauma center for 1 to 16 days. Data are given for the luminal and extraluminal surfaces, each divided into three (female) or four (male) axial locations from internal (bladder) to external (exposed to air), as well as for urine samples for a subset of subjects.

| Subjects | Extraluminal | | | | Intraluminal | | | | Urine |
| --- | --- | --- | --- | --- | --- | --- | --- | --- | --- |
|  | Bladder(1) | Deep urethral (2) | Superficial urethral (3) | External (4) | Bladder (1) | Deep urethral (2) | Superficial urethral (3) | External (4) | Urine |
| Ma1 | 1020 | 2,010 | 2,970 | 8,000 | 9,100 | 8,740 | 11,200 | 8,880 | 29 |
| Me1 | 360 | 70 | 1,840 | 18,100 | 640 | 70 | 120 | 3,700 | 0 |
| Fa2 | 23,000 |  | 28,000 | 47,000 | 35,400 | 46,500 |  | 89,000 | 1,033 |
| Mc2 | 1,910 | 1,450 | 20,000 | 50,000 | 310 | 40 | 430 | 2,090 | 0 |
| Fb4 | 1,110 |  | 1,380 | 9,000 | 0 | 600 |  | 10 | N/A |
| Md5 | 1,830 | 610 | 9,240 | 37,810 | 630 | 290 | 2,210 | 1,200 | N/A |
| Fc9 | 25,600 |  | 7,800 | 20,000 | 5,600 | 3,100 |  | 1,500 | 105 |
| Mb16 | 30,000 | 364,000 | 163,400 | 1,810,000 | 322,000 | 66,000 | 42,000 | 120,000 | N/A |

**Figure Legends**

**Fig. S1**. OTU richness in intraluminal and extraluminal surfaces over time for the *Hha* I (A) and *Msp* I (B) digests. Each point represents the mean and standard error over the length of the catheter for each surface of each individual. Lines indicate a significant or nearly significant relationship; for P values from mixed model analysis of both digests see Table 2. Urinary catheters from 5 males and 3 females catheterized in a level 1 trauma center for 1 to 16 days.

**Fig. S2.** Non-metric multi-dimensional scaling (NMDS ) ordination of the OTU community from the *Hha* I digest based on Bray-Curtis dissimilarity index. Degree of shading indicates the length of time the catheter was in place, white =1 day to black = 16 days. Males are represented by squares and solid lines, and females by circles and dashed lines. I is the intraluminal surface, E is the extraluminal surface, and U is urine. Lines connect samples from the same subject. For each subject, all samples from intraluminal and all samples from extraluminal surfaces were combined. Note that samples from catheters that were in place for longer periods of time are clustered and have lower variability compared to samples from catheters in place for shorter periods of time (significant effect of time, Table 2). Also note that the urine samples tend to be more similar (closer) to the intraluminal samples than the extraluminal samples from each subject. Digest of microbial communities from urinary catheters from 5 males and 3 females catheterized in a level 1 trauma center for 1 to 16 days. Samples from the *MSP* I digest showed similar patterns.

Fig. 1S


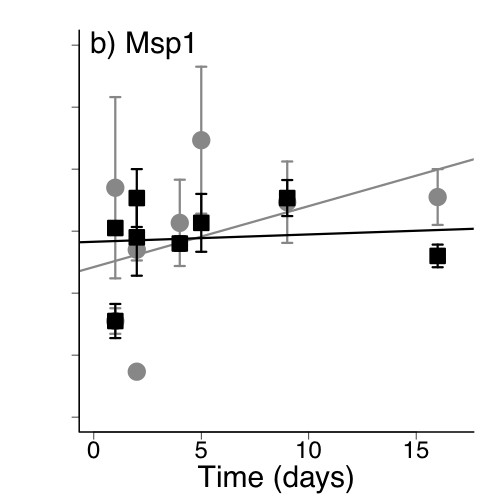

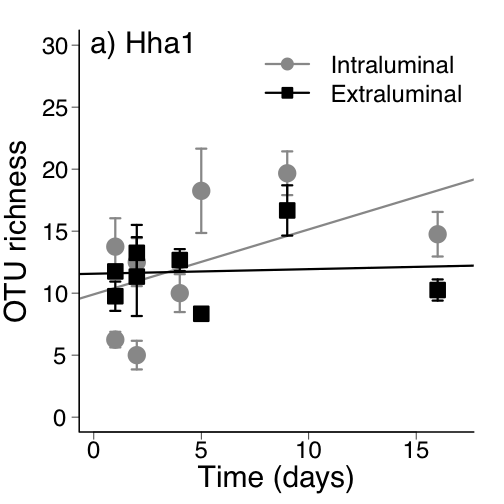


A

Hha1

Msp1

B

Figure S2


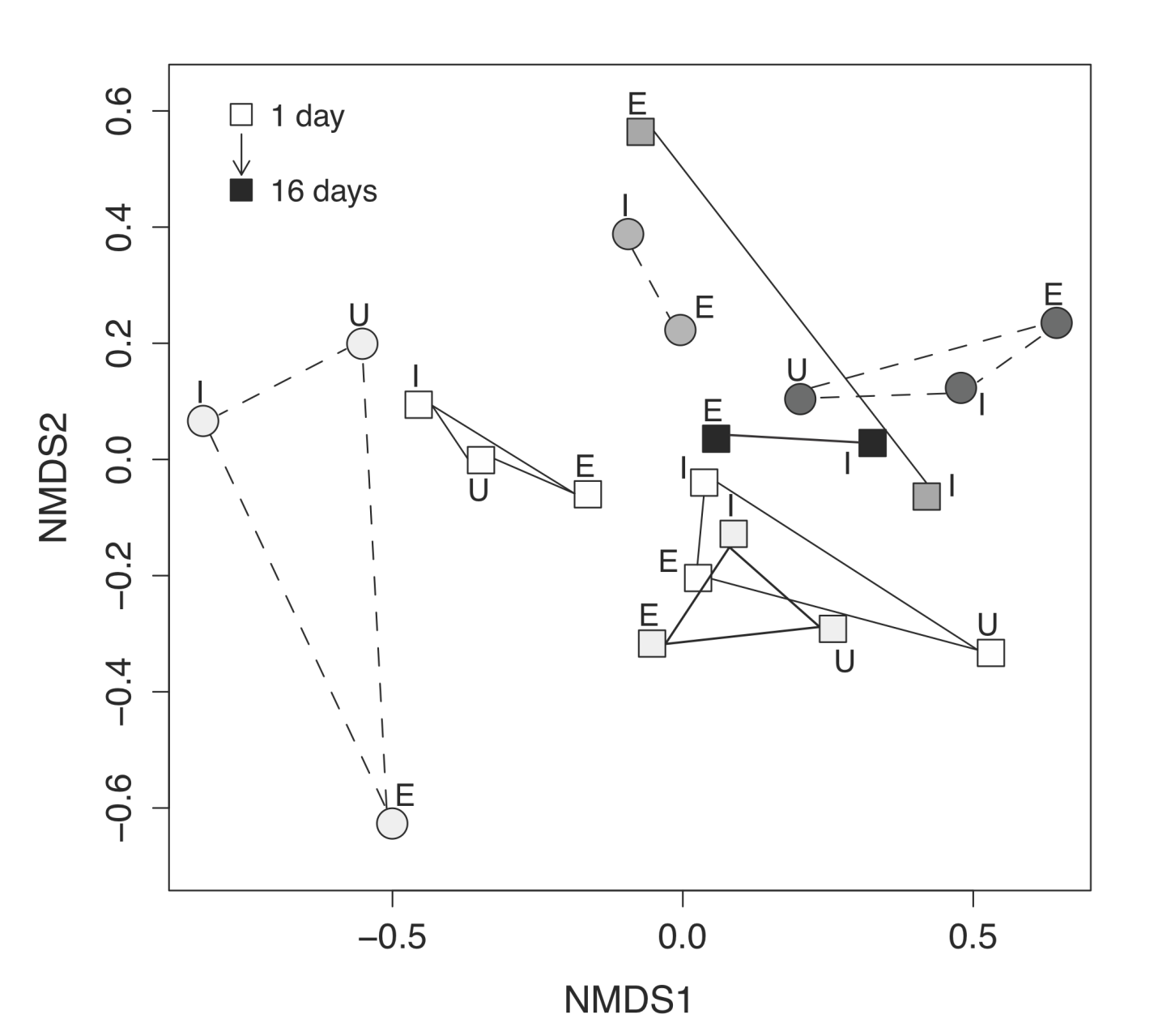

Supplement: Additional file 1 — Table S1. Cultivable bacteria on each axial location of urinary catheters (CFU/part or CFU/ml for urine samples). Urinary catheters from 5 males (labeled Ma to Me) and 3 females (labeled Fa to Fc) catheterized in a level 1 trauma center for 1 to 16 days. Data are given for the luminal and extraluminal surfaces, each divided into three (female) or four (male) axial locations from internal (bladder) to external (exposed to air), as well as for urine samples for a subset of subjects. Figure S1. OTU richness in intraluminal and extraluminal surfaces over time for the Hha I (A) and Msp I (B) digests. Each point represents the mean and standard error over the length of the catheter for each surface of each individual. Lines indicate a significant or nearly significant relationship; for P values from mixed model analysis of both digests see Table 2. Urinary catheters from 5 males and 3 females catheterized in a level 1 trauma center for 1 to 16 days. Figure S2. Non-metric multi-dimensional scaling (NMDS) ordination of the OTU community from the Hha I digest based on Bray-Curtis dissimilarity index. Degree of shading indicates the length of time the catheter was in place, white = 1 day to black = 16 days. Males are represented by squares and solid lines, and females by circles and dashed lines. I is the intraluminal surface, E is the extraluminal surface, and U is urine. Lines connect samples from the same subject. For each subject, all samples from intraluminal and all samples from extraluminal surfaces were combined. Note that samples from catheters that were in place for longer periods of time are clustered and have lower variability compared to samples from catheters in place for shorter periods of time (significant effect of time, Table 2). Also note that the urine samples tend to be more similar (closer) to the intraluminal samples than the extraluminal samples from each subject. Digest of microbial communities from urinary catheters from 5 males and 3 females cathete [file 1756-0500-5-332-S1.doc]
